# Targeting FMRP: A new window for cancer immunotherapy

**DOI:** 10.1002/mco2.233

**Published:** 2023-03-21

**Authors:** Yaguang Zhang, Tong Wu, Junhong Han

**Affiliations:** ^1^ State Key Laboratory of Biotherapy and Cancer Center, and Frontiers Science Center for Disease‐Related Molecular Network West China Hospital Sichuan University Chengdu P. R. China; ^2^ Research Laboratory of Cancer Epigenetics and Genomics West China Hospital Sichuan University Chengdu P. R. China

## Abstract

- FMRP is regulated by Myc and is highly expressed in a variety of human and mouse tumor tissues.
- FMRP recruits Treg and M2 macrophages to form an immunosuppressive tumor microenvironment by IL3, PROS1 and exosomes.
- FMRP‐KO up‐regulates tumor cell secretion of CCL7, which directly activates and recruits CD8+ T cells.
- FMRP‐KO recruits CCR5 and CXCR4 receptor‐positive CD8 T cells indirectly by promoting M1 macrophages to secrete CCL5, CXCL9, and CXCL10.

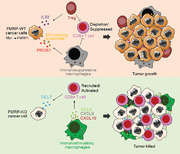

1

CD8+ T cell depletion is the key issue in tumor immunotherapy. Immune checkpoint inhibitors (ICIs) have shown clinical activity in several types of cancer since 2011.^1^ However, some patients have innate resistance to ICI and some develop adaptive resistance to ICI.^2^ There is an urgent need to discover new molecular mechanisms of tumor immune escape to develop new immunotherapy methods. A recent study published in *Science* by Zeng et al. described a novel component, fragile X mental retardation 1 protein (FMRP), that can mediate tumor immune escape, which provides a new insight for immunotherapy (Figure 1).^3^

**FIGURE 1:**
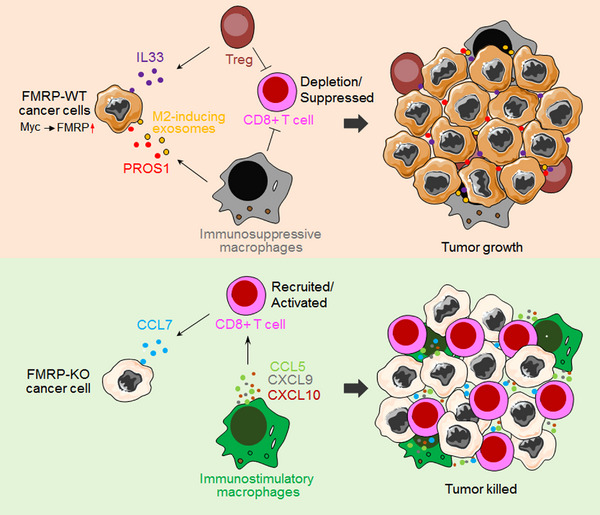
Schematic diagram of FMRP regulating cancer cells immune evasion. Hyperexpression of fragile X mental retardation 1 protein (FMRP) activates Treg cells to inhibit CD8+ T cell activity, cancer cells secret PROS1 and exosomes to induce M2 macrophages, creating a defensing immune desert for tumor (upper). By contrast, FMRP knockout (KO) cancer cells excrete CCL7 to recruit and activate CD8+ T cell while promote CCL5, CXCL9, and CXCL10 secretion to create a T lymphocyte‐activated microenvironment to kill tumor cells (lower).

Highly expressed FMRP in neural cells plays a vital role in proper synaptic plasticity and architecture. FMRP inactivation by a triplet nucleotide repeat expansion leads to a neurodevelopmental disorder called fragile X syndrome.^4^ Recently, several studies have described FMRP new functions involved in cancer invasion and metastasis.^5^ However, whether FMRP functions in immune regulation in solid tumors remains elusive. Zeng et al. demonstrated the role of FMRP in adaptive immunity for the first time. Authors found high expression of FMRP in most tumors detected using tumor tissue microarrays. The similar results were also observed in mouse spontaneous tumors including pancreatic cancer, colon cancer and breast cancer. Subsequently, by transplanting FMRP knockout (FMRP‐KO) and wild‐type (WT) cancer cells into immunocompetent (SCID/beige or NSG) and immunodeficient (FVB/N or Balb/c) mice, they found that FMRP‐KO has not obvious effect on tumor cell growth in vivo and mouse survival in immunodeficient mice. Interestingly, FMRP‐KO significantly inhibited tumor cell growth and improved survival in immunocompetent mice. This suggests that FMRP itself may not be involved in tumor growth regulation, but rather in the immune escape of tumor cells.

By single cell sequencing WT and FMRP‐KO pancreatic ductal adenocarcinoma tissues, authors revealed significant increase in the abundance of effector CD8 T cell subtypes and significant decrease in the abundance of CD4 regulatory T cells (Tregs) in FMRP‐KO tumors. Moreover, they intuitively observed that FMRP‐KO tumor is highly infiltrated by CD8 T cells, and T cell depletion has no effect on WT tumor growth, survival, and anti‐PD‐1 therapy. However, by treating mice with anti‐CD8 or anti‐CD4 antibodies, FMRP‐KO showed significant reduction of CD8 T cells in the blood, strong tumor growth, short survival and the worse therapeutic effect of anti‐PD‐1. Additionally, FMRP KO or knockdown enhanced the ability of CD8 and CD4 T cells to kill tumor cell measured by in vitro co‐culture killing experiments.

Next, CellChat (cell‐to‐cell communication) analysis revealed that FMRP‐KO cancer cells have a higher number of cells‐cell communications with other cell types in the TME compared to WT cancer cells. Among the upregulated ligands in the scRNA‐seq dataset, only C‐C motif chemokine ligand 7 (CCL7) was remarkably upregulated in both mRNA and secreted protein in cultured FMRP‐KO cancer cells. To evaluate the potential of FMRP‐KO cancer cells to inhibit tumor growth through upregulation of CCL7, they established a mouse xenograft model of *Fmr1* and *Ccl7* double‐gene knockout cells (*Fmr1^−/‐^ Ccl7^−/−^*), and found an increase in tumor volume and weight, but a decrease in CD8T cells in *Fmr1^−/‐^ Ccl7^−/−^* versus FMRP‐KO. Meanwhile, the authors also observed the moderate reduction of Tregs number in *Fmr1^−/‐^ Ccl7^−/−^* group, but no change in the expression of tumor‐associated macrophages (TAMs) markers involved in immunosuppression, indicating that CCL7 was only involved in the regulation of inflammation by T cells. In addition, authors found that FMRP‐KO significantly down‐regulates the expression of IL33, a factor is known to stimulate Treg production, while overexpression of IL33 in FMRP‐KO cancer cells promotes tumor growth in mice.

Macrophage is one of major immuno‐modulatory factors in tumor. The authors found that WT tumor cells can induce bone marrow‐derived macrophages (BMDM) polarization to M2‐like macrophages, mainly by upregulating the mRNA of *Arg1*, *Il10*, *Adm* and *Cd163* immunosuppressive genes in M2‐like TAMs. Meanwhile, they also demonstrated high expression of Pros1, which inhibits the polarization of M1‐like macrophages, and an exosome‐associated gene Cd63 in WT versus FMRP‐KO cancer cells analyzed by RNA‐seq and scRNA‐seq. The effect of FMRP‐KO on suppression of immunosuppressive ARG1 expression was inhibited in M1‐polarized BMDM and WT versus FMRP‐KO co‐cultured cells treated with PROS1 recombinant protein. When PROS1 is overexpressed in FMRP‐KO cancer cells, the upregulation of PROS1 promotes tumor growth and reduces the expression of CD86 in TAM. In addition, WT cancer cells could secrete more exosomes to activate the expression of *Arg1*, *Il10*, *Adm*, and *Cd163* genes. In contrast, FMRP‐KO cancer cells could recruit CCR5^+^ and CXCR4^+^ CD8 T cells to indirectly kill cancer cells by promoting M1 macrophages to secrete proinflammatory chemokines CCL5, CXCL9, and CXCL10.

Mechanically, authors reveal that the high expression of FMRP in tumors is not related to mutation and amplification of the FMRP gene but is directly regulated by transcription of the proto‐oncogene MYC by analyzing the TCGA patient cohort, the ENCODE database and transcription factors. As an RNA collection protein, FMRP broadly regulates protein translation and mRNA stability. Cross‐linking immunoprecipitation sequencing (CLIP‐seq) analysis shows the direct association of FMRP with *Ccl7* mRNA, implying that FMRP depletion may abolish the inhibition of *Ccl7* translation in cancer cells, which in turn directly activate CD8 T cells. Similarly, FMRP as a translational regulator promotes the production of immunosuppressive macrophages by directly binding to Pros1 mRNA. By establishing a 156‐gene transcriptional regulatory network for FMRP, authors indicate the potential of this network in evaluating CD8 T cell infiltration and prognosis in patients.

Taken together, this outstanding work by Zeng et al. reveals a novel mechanism involved in FMRP regulation of immune evasion in cancer. Briefly, upregulated FMRP in tumor cells could activate Treg cells by binding to the immune‐associated gene mRNA, rejecting and inhibiting CD8 T cell activity. Moreover, PROS1 ligands and exosomes induce M2‐like macrophages to jointly create an immunosuppressive microenvironment. In contrast, tumor cells with low or absent FMRP expression promote the secretion of CCL5, CXCL9, and CXCL10 from macrophages to create an immune activation microenvironment in cooperation with CCL7. In this paper, Zeng et al. focus on exploring the essential role and molecular mechanism of FMRP in tumor communication with T cells and TAMs. However, whether FMRP is involved in the crosstalk between tumor cells and other cells in the tumor microenvironment is not clear.

Since upregulation of FMRP is found in a variety of cancers, and FMRP KO increases the infiltration of CD8+ T cells in a variety of tumors, suggesting that aberrant FMRP in different types of cancers may involve the same immune escape mechanism. Accordingly, targeting FMRP to activate CD8 T cell immune activity may be a promising pan‐cancer therapeutic strategy. Therapeutic strategies for targeting FMRP include, but are not limited to, the development of small molecule inhibitors, proteolytic targeting chimera, antibody‐drug coupling, peptide‐drug coupling and nanomedicine, etc. Based on Zeng et al.’s finding, targeting FMRP in combination with the treatment of anti‐PD‐1 or anti‐PD‐L1 may lead to better therapeutic outcomes. However, what concerns us is whether FMRP‐targeted therapy can achieve the same satisfactory effect as in mice mentioned in this article due to the high heterogeneity of tumor patients. In addition, how to bypass FMRP neuron‐specific irreversible damage also need further exploration. Undoubtedly, this work inspires us that there are great opportunities to uncover more immune‐related and highly expressed genes in tumors and to address acquired resistance to immunotherapy by targeting specific immune‐related genes.

## AUTHOR CONTRIBUTIONS

Y.Z. and T.W. contributed equally to this work. Y.Z. and T.W. conceived and drafted the manuscript. Y.Z. drew the figure. J.H. provided valuable discussion and revised this manuscript. All authors have read and approved this manuscript.

## CONFLICT OF INTEREST STATEMENT

The authors declare no conflict of interest.

## ETHICS STATEMENT

Not applicable.

## Data Availability

Not applicable.
